# Dictionary of Medicine, or General Repertory of the Medical Sciences, Considered Theoretically and Practically

**Published:** 1845-04

**Authors:** 


*Dictionnaire de Médecine ou Repertoire Général des Sciences Médicales considérées sous les Rapports Théoretique et Pratique.* Par ADELON, BÉCLARD, &c. &c. 2ème Edit. Tome XXIXème. SUC—TYP. 8vo. pp. 884. Paris, 1844.

*Dictionary of Medicine, or General Repertory of the Medical Sciences, considered theoretically and practically.* By MM. ADELON, BÉCLARD, &c.

The first two works, the titles of which are given above, are on kindred departments of medicine. The last, of which we have before spoken most favorably, is intended, and expected, when complete, to embrace the whole circle of the medical sciences.

The treatise of MM. Hardy and Behier is the production of young men, hitherto not much known in the profession; but not necessarily, on that account, the less estimable. In their preface they modestly apologize for a defect—if it be one, which we hope will annually diminish with them—their want of distinction. It is their first work; and it is "dedicated to the memory" of M. Biett; "as a feeble testimony of profound gratitude and poignant regrets." p. xv.

The work needs no apology. It is carefully written, and contains a more complete account of the received opinions on general pathology amongst the countrymen of the authors than any work with which we are acquainted; and if translated, with additions, by a competent person, of the views of observers, who speak the Teutonic language in all its varieties—and in it we include the English—it would be a valuable accompaniment to the student here; and might be consulted with much advantage by the practitioner.

After some clear and concise views on disease, it investigates, in detail, etiology; symptoms and signs; progress of diseases; termination of diseases; seat of diseases; pathogeny; nature of diseases; *delimitation* or pathological individuality of diseases; diagnosis; prognosis; semeiological examination of the morbid signs furnished by different apparatuses,—and lastly, treatment of diseases or general therapeutics.

This is but the first volume of the work; and we fervently hope, that the authors have received sufficient encouragement to persevere in their course. At the same time, the competition in works on internal pathology in France is now considerable; and many will, doubtless, think those of Valleix and Grisolle preferable to the one we are noticing.

The treatise of M. Grisolle consists of two very large octavo volumes, and embraces the whole domain of internal pathology. The name and previous productions of the author are well known among us. We wish we could say, that he was as well acquainted with what emanates from English and American writers. He seems, indeed, to know little, or—if he knows more—to care little about any thing that does not reach him in a French dress; in this respect resembling too many among us. We have often been impressed with the *cosmopolitan* knowledge possessed by our best informed writers, compared with those in France, even now, when the intercourse between that country and the numerous people who speak the English language has become so intimate and constant. Any one who will compare works that issue from the American press with those of France, or even of England, must be struck with their signal difference in this respect. It need scarcely be said, that in a science of observation and reflection like medicine we are bound neither to discard nor to slight the testimony of respectable observers anywhere.

M. Grisolle dedicates his work to M. Chomel; his "very dear master;" who gave him, he affirms, the idea of it; and in the preface he thus "defines his position:" "A stranger to every species of sect; independent by disposition as by position, I have neither had to defend nor attack, against my conscience, any man or any doctrine: every where and always I have sought truth with the impartiality of the historian, and have proclaimed it whencesoever it may have come. If, notwithstanding, I have omitted to cite some important works, I beg my *confrères* to ascribe this to mere forgetfulness, and in no manner to regard it as an act of ill feeling, or of a desire to do wrong." p. viii.

The classification adopted by him, for which he apologizes, and not, we think, without cause, is the following: 1, fevers; 2, diseases consisting in a *vice* of proportion in the blood; 3, inflammations; 4, morbid secretions; 5, poisonings; 6, lesions of nutrition; 7, organic transformations and accidental morbid products; 8, neuroses; and 9, diseases special to certain organs. The last class signally exhibits the imperfection of the classification. For example, amongst the "diseases special to the digestive organs," we have—the first dentition and the accidents that complicate it—indigestion—*embarras gastrique; embarras intestinal*—constipation—intestinal invagination or volvulus, and diabetes. [?] Amongst the special diseases of the liver, but one—jaundice! Of the special diseases of the heart, but two—insufficiency of the valves of the heart, and sanguineous concretions in that organ! Matters of classification, however, we have never thought of essential importance. Still, if an individual leaves those arrangements that are commonly received and adopts another, we have a right to expect some apparent, if not real, improvement. Such is not the case here.

M. Grisolle has touched on almost all the maladies that belong to internal pathology: in this respect he is full; but when we examine the individual articles, we cannot accord them the same compliment. The size of the work would of course preclude great detail; but the articles are not equal. Some, on which he had already written at length, have naturally, perhaps, more space allotted to them than others.

As we remarked of the work of MM. Hardy and Béhier, so we may say, in the main, of that of M. Grisolle. It conveys, in epitome, the received opinions of the author's countrymen; but in this instance, chiefly on special pathology and therapeutics. His "first class of diseases" comprises fevers; and it is interesting for us to inquire into the opinions of a pupil of Chomel; and a "titular member of the Medical Society of Observation," on this—to us, and to every one—interesting class of diseases.

His first genus of fevers is continued fever, in which he includes ephemeral fever, inflammatory fever, typhoid fever, typhus fever of England, bilious fever of hot climates, yellow fever, and plague; thus classing together, amongst others, the continued and remittent fevers. But we shall permit the author himself to speak on this matter.

"The consideration of the type of fevers serves to establish a very natural and important division, found even in the Hippocratic books. Thus, if the febrile movement continues without interruption, the fever is said to be *continued;* if it ceases, to recur at regular intervals, it is then *intermittent;* if when of the continued form, it presents periodical exacerbations, it is called *remittent.* In all times, physicians have distinguished several species of continued fever, and have especially founded their divisions on the predominant characters, or on the idea, which they formed on the causes and nature of fever. Hence have originated the divisions of continued fever into adynamic and ataxic, inflammatory, bilious, mucous, putrid, malignant, &c. These arbitrary divisions, with which Pinel especially, overloaded pyretology, have given a retrograde impulse to science, and arrested the tendency, which existed since Chirac, to the fusion of continued fevers. Pinel exerted such an empire over his contemporaries, that MM. Petit and Serres having in 1813 described the intestinal lesions that are characteristic of typhoid fever, believed that they had discovered a new disease, essentially distinct from the adynamic and ataxic fevers of Pinel, to which they assigned a separate existence. Broussais, who came a few years afterwards,—impelled by an instinctive feeling, rather than by conviction, the result of exact observation,—endeavoured to effect the fusion of continued fevers, and placed them in the digestive tube, as the term *gastro-entérite,* which he gave them, demonstrates. But this term was itself erroneous, for it supposed a gastritis, which rarely exists. Moreover, under this denomination Broussais confounded several different diseases, without, however, seizing hold of the distinctive characters of typhoid fever. Such was the state of the science, when in 1829 appeared the researches of M. Louis, who forthwith established the fusion of all the grave continued fevers of this climate into one only—typhoid fever. The demonstration of this grand truth is unquestionably one of the most beautiful conquests of contemporary medicine: it has effected a total revolution in the old pyretology, since it has demonstrated, that grave continued fevers, so different in appearance, considered by every one as distinct maladies were at the bottom, and in their nature identical; constituting but one affection, which might appear, according to circumstances under varied forms. (Chomel and Genest.) Typhoid fever, however, comprises only the grave forms of pyrexia, formerly admitted, and to complete the knowledge of the continued fevers of this climate, other species must still be admitted—and these are *ephemeral* and *inflammatory* fevers. But in some other regions of the globe, typhoid fever does not comprise every grave form of fever observed there; thus, the *typhus fever of Ireland*, *yellow fever*, the *bilious fever* of hot climates, and the *plague* of the east, are special continued fevers, distinct from the typhoid affection." p. 8.

This division will of course not satisfy those who believe, that all fevers are either typhoid or typhus, and who affirm, that if there be such a fever as the inflammatory, they have never seen it. In our last number we showed, that Louis, Chomel and De Claubry unhesitatingly admit the *identity* of typhus and typhoid in France; and now we have M. Grisolle boldly affirming that the typhus or *Peste d'Europe*, the synonymes of which are—to use his own language—"*Fièvre pestilentielle*, *typhus contagieux*, *fièvre de Hongrie*, *des hôpitaux*, *des camps*, *des prisons*, *fièvre pétéchiale lenticulaire*, &c., are one and the same affection—typhoid fever, whilst "the continued fever of England or typhus fever," he thinks—founding his views on the observations of one or two gentlemen, not English physicians, whom he cites—is a distinct malady. It consequently appears, that many of the pathologists of France, who have been so much occupied with the diagnosis, &c. of typhoid fever, and have thrown so much light on the subject, believe it no longer to be distinct from the typhus of France and of Europe. *Now* they have no typhus in continental Europe; all is typhoid. Typhus, distinct from typhoid, is confined, according to them, to England, and is sometimes seen here. This view is not, however, embraced by all. In the article Typhus of the *Dictionnaire de Médecine*, just received, and now before us, M. Dalmas maintains the distinct character of the typhus and the typhoid fevers of the European continent

"At the present day," he observes, "it is agreed to take this word [typhus] in a much more restricted sense; and in this place, with Hildenbrand, Pinel and J. Frank, we shall understand by typhus but one disease, that which, amongst other names, has received those of *camp fever*, *hospital fever*, and *jail fever*. It is to this affection—in our view, one and very distinct—that we give the name typhus, and we refuse it to every other. We separate, consequently, typhus from plague, cholera, and yellow fever, which, in our opinion at least, differ from it in an infinity of respects. We separate it also from typhoid fever, or from *dothienentérie*, properly so called; not that we are unaware of the analogies that exist between them, but precisely because these analogies seem to us to be sufficiently indicated by the happy expression—*typhoid fever*, and because it does not appear to us necessary to push the approximation further."

The typhoid and typhus fevers of France are thus considered by M. Dalmas to be only *analogous;* whilst by MM. Louis, De Claubry, Chomel and Grisolle, they are esteemed *identical.*

The typhus fever of the English, M. Dalmas believes, resembles greatly the typhus of armies, which, as we have shown, M. Grisolle considers to be the same as typhoid fever. "It resembles it," says M. Dalmas, "by its causes, symptoms, and the alterations revealed by cadaveric examinations. The differences, for there are some, and we do not pretend to deny it, are only, in our opinion, secondary, and are owing to the mode of action of the causes. This mode of action is much more slow in the bosom of the population of which we speak than in camps. Armies are exposed to vicissitudes and to influences incomparably more rough (*brusques*); but at the bottom, the effects are of the same nature, and do not constitute morbid conditions essentially different." p. 841.

In support of his view, that the typhus of the English ought not to be confounded with the typhoid fever of the French, he cites testimony on which, he says, he reposes every confidence, that of Professor Alison, of Edinburgh, and of Dr. Gerhard, of this city, "who, after having observed the typhoid fever at Paris, studied the typhus fever of the English." We know not where M. Dalmas obtained his knowledge of the opinions of Professor Alison. We have always regarded him as maintaining the view adopted by ourselves, that the common continued fever of England is an adynamic pyrexia,—at times accompanied by the intestinal lesion; at others not. In his "*Outlines of Pathology*," (Amer. edit., p. 239, Philada., 1844,) Professor Alison thus expresses himself:

"But the most common abdominal affection in fever in this climate is that in which the intestines are chiefly concerned, which comes on at different times, but chiefly, and with most danger, in the later stages; and this is marked sometimes by severe pain and tenderness, but in general, chiefly by diarrhœa, seldom violent, but often obstinate: it is attended with occasional griping, (often aggravated by ingesta,) sometimes with scanty mucous and bloody stools and tenesmus, as in dysentery, but more frequently without such symptoms. It is often accompanied with gradually increasing tympanitic distention, and often occasions long protraction of the typhoid febrile symptoms, in the course of which great emaciation, extreme debility, and often a peculiar dryness of the skin are observed; and in a few cases it is followed either by sudden exhausting hæmorrhage, or by a sudden attack of acute pain and tenderness of abdomen, with vomiting and rapid sinking.

"It is well ascertained that such abdominal symptoms in fever depend very generally on inflammation of the mucous membrane, and especially of the mucous glands of the intestines; but this inflammation tends rapidly to ulceration; and that the attacks of sudden hæmorrhage, or of acute pain and tenderness in the advanced stages of such cases, usually depend on erosion of blood vessels or perforation of the whole coats, and escape of the contents of the intestines into the cavity of the abdomen, in the course of that ulceration; but that inflammation commencing in, or confined to, the peritoneum, is equally rare in fever, as that of the serous membranes within the chest."—

And in treating of the morbid appearances observed in continued fever in the glands of Peyer and Brunner, he says:—"Such unequivocal disease of the mucous membrane of the alimentary canal is found, either alone, or in combination with the other diseased appearances above mentioned, in a majority of the fatal cases of continued fever, which occur in Paris, and *in a large proportion of those that occur in London*; but in a much smaller proportion of fatal cases in Scotland. They are decidedly more frequent, at least in this country, after the fevers of children and young persons than in advanced life, and in fevers that are fatal at an advanced period than those that are rapidly fatal."

On the subject of the identity or unity of the continued fevers of Great Britain, Professor Alison speaks in the most unequivocal language. After describing the different phases which continued fever may assume, he concludes:—

"But although we consider it of real importance to mark these distinctions, both in the essential symptoms of fever itself, and also in the concomitant local affections which distinguish individual cases, and sometimes epidemics, from each other, yet, it appears equally certain, from consideration of the nature of these distinctions, of the manner in which the varieties, thus marked, graduate into one another, or are blended together in the same cases, and by the many varieties, which present themselves in the same epidemic, and in immediate connexion with each other, *that all the continued fevers of this climate must be regarded as fundamentally the same disease.*"

Surely these views cannot be regarded by M. Dalmas as confirmatory of his own!

It is evident to us, that this whole subject is becoming more and more illuminated; and that many of the researches of late years have rather tended to involve it in obscurity.

We have been led into these remarks in consequence of those in which we indulged in the last number, and of the strong ground assumed by M. Grisolle on the subject.

It would be impossible to give any adequate idea of the manner in which M. Grisolle treats of the various diseases investigated in his extensive *tomes.* The course commonly pursued by him is to take up in succession the consideration of anatomical characters, symptoms, diagnosis, prognosis, etiology, and treatment.

Under the head of Delirium Tremens we observe the following remarks:—

"Esquirol, Georget, and recently M. Calmeil have advised nothing more than a mild, almost expectant treatment in delirium tremens. In simple cases, M. Calmeil only prescribes sweet drinks and tepid bathing. If the tongue be coated, he gives an emetic; if there be constipation, he purges; and if signs of inflammation or congestion towards the brain appear, he combats them by bleeding, which is rarely necessary in the disease. This skilful physician does not prescribe opium, imitating in this the practice of M. Esquirol, whose patients were commonly cured in three days by simple expectation." p. 62.

It is obvious, that on this disease also, M. Grisolle is profoundly ignorant of views promulgated, and acted upon with success, elsewhere. We cite the observation, however, to show that individuals of extensive experience have found—what we have long maintained—that neither the opium nor the alcoholic treatment can be often necessary; and that there is perhaps no case in which we are wholly justified in administering either to the extent that is sometimes done. The numerical method has, we think, sufficiently settled, that neither of these is *the most* advisable system of medication.

Of the *Dictionnaire de Médecine,* we need but reiterate the favourable opinion we expressed in a former number of the "Examiner." It has already exceeded its proposed limits—twenty-five volumes; but will probably be concluded in one or two volumes more. It is a most valuable addition to a medical library.

